# A Web- and Mobile-Based Map of Mental Health Resources for Postsecondary Students (Thought Spot): Protocol for an Economic Evaluation

**DOI:** 10.2196/resprot.9478

**Published:** 2018-03-29

**Authors:** Amandeep Kaur, Wanrudee Isaranuwatchai, Aliya Jaffer, Genevieve Ferguson, Alexxa Abi-Jaoude, Andrew Johnson, Elisa Hollenberg, David Wiljer

**Affiliations:** ^1^ The Western Centre for Public Health and Family Medicine Western University London, ON Canada; ^2^ Institute of Health Policy, Management and Evaluation University of Toronto Toronto, ON Canada; ^3^ Centre for Excellence in Economic Analysis Research St. Micheal's Hospital Toronto, ON Canada; ^4^ Office of Education Centre for Addiction and Mental Health Toronto, ON Canada; ^5^ Department of Psychiatry University of Toronto Toronto, ON Canada; ^6^ Education Technology and Innovation University Health Network Toronto, ON Canada

**Keywords:** economic evaluation, health economics, transition-aged youths, participatory action research, mental health

## Abstract

**Background:**

Youth demonstrate a low propensity to seek help for mental health issues and exhibit low use of health services despite the high prevalence of mental health challenges in this population. Research has found that delivering interventions via the internet and mobile devices is an effective way to reach youth. Thought Spot, a Web- and mobile-based map, was developed to help transition-aged youth in postsecondary settings overcome barriers to help-seeking, thereby reducing the economic burden associated with untreated mental health issues.

**Objective:**

This paper presents the protocol for an economic evaluation that will be conducted in conjunction with a randomized controlled trial (RCT) to evaluate the effectiveness and cost of Thought Spot compared with usual care in terms of self-efficacy for mental health help-seeking among postsecondary students.

**Methods:**

A partially blinded RCT will be conducted to assess the impact of Thought Spot on the self-efficacy of students for mental health help-seeking. Students from 3 postsecondary institutions in Ontario, Canada will be randomly allocated to 1 of 2 intervention groups (resource pamphlet or Thought Spot) for 6 months. The economic evaluation will focus on the perspective of postsecondary institutions or other organizations interested in using Thought Spot. Costs and resources for operating and maintaining the platform will be reported and compared with the costs and resource needs associated with usual care. The primary outcome will be change in help-seeking intentions, measured using the General Help-Seeking Questionnaire. The cost-effectiveness of the intervention will be determined by calculating the incremental cost-effectiveness ratio, which will then be compared with willingness to pay.

**Results:**

The RCT is scheduled to begin in February 2018 and will run for 6 months, after which the economic evaluation will be completed.

**Conclusions:**

We expect to demonstrate that Thought Spot is a cost-effective way to improve help-seeking intentions and encourage help-seeking behavior among postsecondary students. The findings of this study will help inform postsecondary institutions when they are allocating resources for mental health initiatives.

**Trial Registration:**

ClinicalTrials.gov NCT03412461; https://clinicaltrials.gov/ct2/show/NCT03412461 (Archived at WebCite at http://www.webcitation.org/6xy5lWpnZ)

## Introduction

### Background

According to the National Alliance on Mental Illness [1], approximately 75% of mental illnesses develop by age 24. Additional evidence suggests that the first onset of mental disorders usually occurs between ages 18 and 24, which is the typical age range of postsecondary students [2].

Transition-aged youth in postsecondary school can face major barriers to academic success as a result of mental illness. Languishing mental health or the presence of mental illness can impair their ability to function [3]. Mental health challenges may adversely affect academic performance and educational attainment by reducing learning opportunities and decreasing the ability to absorb information and demonstrate learning [4,5]. Eisenberg and colleagues [6] found that college students with depression had lower productivity rates in school compared with students without the disorder [6]. This lower performance increases the risk of lower grade point averages and course or semester withdrawal.

Early onset of mental health issues, such as depression, is also associated with the early termination of schooling [4,7]. Adolescents with anxiety disorders are at increased risk of academic underachievement and school dropout compared with adolescents without these disorders [8]. Disruptions to educational attainment can have a wide range of effects on physical and mental health in adulthood [6,9] and on social ability [10]. They also reduce productivity and create large economic losses for the individual [5,11,12]. Students, who are unable to keep up with academic expectations and are thus at increased risk of withdrawing from the semester, may lose investments in their education in the form of nonrefundable tuition payments and lost future income due to delayed graduation and later entry into the workforce [13,14]. Moreover, disrupted education limits career prospects [5].

Mental health issues among students also have a large economic impact on postsecondary institutions [15]. The need for mental health services in colleges and universities has increased the financial burden on these institutions. Canadian postsecondary institutions continue to face challenges associated with limited resources and funding due to the greater costs associated with the high prevalence of mental health issues in young people [15].

### Mental Health Climate in Canadian Postsecondary Institutions

The burden of mental illness and emotional crisis among students has been increasing at an exponential rate across Canadian postsecondary institutions: schools are seeing a 200% increase in the demand for counselling services and more than 50% of students report feelings of hopelessness and anxiety [16]. In an assessment of the mental health climate across 41 Canadian postsecondary institutions in 2016, the American College Health Association found that 58% to 73% of students reported feeling hopeless, overwhelmed, lonely or sad, and 13% had seriously considered suicide in the previous 12 months [17]. Approximately 1 in 10 students reported self-harm, including burning, bruising, and cutting. Moreover, although students indicated that mental health concerns, financial difficulties, gambling, relationship and roommate problems, drugs and alcohol use, and other factors affected their academic performance, less than half reported ever receiving help from a medical or mental health care provider or from on-campus counselling services [17].

Despite the high prevalence of mental health and addiction challenges among postsecondary students, there is a low propensity to seek professional or nonprofessional help, resulting in a low use of health and wellness services [3,18-22]. Barriers to seeking help include stigma and embarrassment, trust and confidentiality concerns, poor mental health literacy, negative attitudes or shame for seeking professional help, and lack of knowledge about where to get help [23-26]. These findings highlight the need for an intervention that helps students overcome barriers to seeking help. With 90% of youth using the internet, health interventions hosted on the Web (electronic health) or on mobile devices have been found to be effective methods for reaching this population [27-30]. In an effort to reach this population of postsecondary students (16-29 years of age), the University of Toronto’s Faculty of Medicine and the Centre for Addiction and Mental Health worked with partners from Ryerson University, the Ontario College of Art and Design, and ConnexOntario to engage with university and college students to co-develop a mobile and online resource called Thought Spot. The project was funded by the Ontario Ministry of Training, Colleges and Universities.

The Thought Spot platform was created through collaboration with postsecondary students. The student-led project aims to improve postsecondary students’ knowledge of, access to, and navigation within addiction and mental health services by digitally mapping mental health, health, and wellness services in the Greater Toronto Area in Ontario, Canada [31]. This Web- and mobile-based map strives to help students overcome barriers to help-seeking, such as health illiteracy and stigma, by increasing their knowledge of local services while simultaneously decreasing the need for intermediaries such as friends, family members, and physicians.

### Objectives

This paper describes a proposed protocol for an economic evaluation of Thought Spot. The evaluation will be conducted using data from a randomized controlled trial (RCT) that evaluates the impact of the Web- and mobile-based app compared with usual care on self-efficacy for mental health help-seeking among university and college students [31]. By conducting an evaluation through an economic lens, researchers will be able to quantify the cost-effectiveness of the intervention. The evaluation will assess the cost and effect of the intervention on mental health help-seeking, and the findings will inform future recommendations regarding intervention use.

## Methods

### Study Design and Target Population

The Consolidated Health Economic Evaluation Reporting Standards guidelines were used to develop this protocol to ensure that the reporting of relevant information for the economic evaluation is consistent with the international standards set out by the International Society of Pharmacoeconomics and Outcomes Research [32].

The economic evaluation will be conducted using data from the primary study, which is a partially blinded RCT with two arms. The full protocol for the RCT has been published elsewhere [31]. The study will recruit 472 students, aged 17 to 29 years, at George Brown College, Ryerson University, and the University of Toronto, who have self-identified as having mental health concerns or an interest in managing their mental health. We will recruit participants through recruitment flyers and messaging, both online (eg, social media and listserv), and physical posters around campus. All participants must have functional competency in English and access to digital devices compatible with the Thought Spot digital platform. Participation is strictly voluntary, and participants may withdraw from the study at any time. Individuals who self-report being actively suicidal will be excluded from the study. Participants will be randomly assigned to the control arm or the intervention arm. The study will take place over 6 months, with measurements taking place at baseline, 3 months, and 6 months. Additional information on the study design and methods is available in the project protocol paper [31].

### Intervention

Participants in the intervention arm will receive access to the Thought Spot platform. They will watch an online “tour the app” video and receive login instructions via email. Participants in the control arm will receive a resource pamphlet via email tailored to each school [31].

### Perspective

The growing need for mental health services for young people has increased the economic burden on postsecondary institutions. A cost-effective intervention for students may reduce the financial strain. To this end, the analysis in this study will take the perspective of potential adopters and promoters of the Thought Spot platform, namely postsecondary institutions and other organizations supporting transition-aged youth.

### Estimating Resources and Costs

The study will report the total resources and costs associated with researching, developing, implementing, and evaluating the Thought Spot platform. These values include costs of starting the project anew rather than costs associated with adopting the Thought Spot project. Because many of the research and development costs will not be relevant to future project adopters, only costs and resources associated with hosting and maintaining the Thought Spot platform will be included in the economic evaluation. Project costs that have already been incurred and that will not be assumed by future project adopters will be excluded in the cost-effectiveness calculations. All costs and effects will be valued using the study period as the time horizon (6 months). All costs will be reported in 2018 Canadian dollars.

### Choice of Health Outcomes

The primary effect variable in the economic evaluation will be change in help-seeking intentions among participants for the duration of the RCT. This change will be assessed using the General Help-Seeking Questionnaire (GHSQ). Responses on this 10-item, 7-point Likert scale range from “extremely unlikely” to “extremely likely” to measure the likelihood that participants will seek help from various formal and informal sources. A higher score indicates greater intent to seek help. The GHSQ scale was chosen to measure the primary outcome because validation studies have suggested a positive and significant correlation between to seek mental health care and seeking care [33]. As a result, an assumption of the evaluation is that secondary outcomes (ie, positive changes in help-seeking behaviors among participants) are attributed to positive changes in the primary outcome, help-seeking intentions.

### Analytic Methods

The evaluation will report the cost per change in help-seeking intentions among the target population. The cost-effectiveness of the intervention will be reported using the incremental cost-effectiveness ratio (ICER) [34], using the following formula:

ICER=ΔC/ΔE

where ΔC represents the difference in cost between the Thought Spot platform and usual care (C_treatment_–C_usual care_), and ΔE represents the change in help-seeking intentions among the intervention and control arms as measured by the GHSQ (E_treatment_–E_usual care_). The ICER will represent the extra cost per additional increase in help-seeking intentions. Cost-effectiveness acceptability curves and 95% confidence intervals will be generated to represent the uncertainty around this value [35].

The calculated ICER can subsequently be compared with willingness to pay (WTP) which allows individuals to value a benefit in terms of a monetary value. WTP allows for recommendations to be made about intervention use by determining whether a decision maker is willing to pay the cost to receive one additional outcome. WTP can be elicited in various ways including directly asking individuals whether they would pay an amount (which is varied) for one additional outcome. However, this WTP amount can be arbitrary and as such may not be entirely accurate. Also, different decision makers may have different WTP values. Thus, in order to ensure this economic evaluation is generalizable across different settings, net benefit regression will be used to vary WTP to evaluate how recommendations about intervention use change based on the estimated ICER [36].

Recommendations about intervention use can be made by comparing the ICER with a threshold WTP. If the ICER value is less than the WTP, the intervention is considered cost-effective compared with usual care. Varying WTP allows researchers to determine how recommendations based on the ICER change. In this way, the incremental net benefit (INB) of the intervention against the expected net benefit of usual care can be calculated as follows:

INB=WTP*ΔE–ΔC

WTP*(E_treatment_–E_usual care_)–(C_treatment_–C_usual care_)

[(WTP*E_treatment_)–C_treatment_]–[(WTP*E_usual care_)–C_usual care_]

=NB_treatment_–NB_usual care_

In this formula, NB represents net benefit, and INB is the additional value in monetary units created by the intervention compared with usual care [36]. By varying WTP using low, medium, and high estimates, a one-way sensitivity analysis can be conducted to assess the cost-effectiveness conclusion to WTP assumptions [36]. This method, net benefits regression, uses regression techniques to yield stronger estimates for cost-effectiveness [36].

Alternative costing scenarios for project adopters will be explored by varying the main incremental cost drivers: the cost of hosting the Thought Spot platform, which varies by the number of end users accessing the platform, as well as the cost of data maintenance (ie, creating, validating and maintaining new spots on the platform). This scenario analysis will demonstrate how the ICER changes as the cost of providing the platform to a greater number of users changes at postsecondary schools.

## Results

Phase 1 of the project ran from September 2015 to December 2017 and informed the redevelopment and optimization of Thought Spot. Phase 2 began in early 2018. It will involve an RCT to evaluate the impact of Thought Spot 2.0 on help-seeking intentions and behavior.

The economic evaluation of Thought Spot will be conducted during Phase 2. Results will be used to report the costs associated with maintaining the Thought Spot platform from the point of view of future project adopters and to report outcomes for postsecondary students associated with using the platform. Ultimately, the evaluation will calculate the cost-effectiveness of the Thought Spot platform in improving health-seeking intentions and behaviors.

## Discussion

### Study Rationale

Research has found that postsecondary students tend to trust Web-based sources for health information and advice, and that they are likely to seek help online first [37,38]. This population reports wanting help in various areas: determining whether they have a mental health problem, finding support, becoming empowered through health information without the assistance of an intermediary, and connecting with peers [39,40]. Thought Spot provides a way for students with mental health concerns to engage in an online community anonymously, access information, and gain awareness of mental health and wellness services in the area. Consequently, we expect Thought Spot to promote help-seeking intentions and encourage help-seeking behavior among postsecondary students.

### Generalizability and Future Research

The availability of resources and funding varies across Canadian postsecondary institutions, which leads to a disparity in mental health services offered to students as part of usual care. This situation limits the generalizability of the study’s cost-effectiveness findings to all postsecondary institutions. To reduce inequity in health services across college and university campuses, a consistent provincial strategy could be developed that incorporates platforms like Thought Spot [15]. As individuals become more likely to seek help, the need for services aimed at improving self-management of mental health issues will increase. Therefore, it is important to simultaneously develop provincial strategies and resources to increase mental health support across campuses.

Thought Spot has the potential to benefit other populations facing similar barriers to mental health help-seeking, including secondary school students and young professionals. Further research would be required to determine the clinical significance and cost-effectiveness of this intervention for other populations. With relatively few studies that have assessed the clinical significance and cost-effectiveness of electronic mental health interventions, the current study will contribute to a growing body of evidence.

### Limitations

A limitation of the evaluation is the assumption that choosing the perspective of postsecondary institutions, rather than a societal perspective, is the best approach. Conducting the evaluation from the viewpoint of potential project adopters excludes costs and benefits of the project to society, but we chose this approach under the assumption that the institutional perspective would include the most relevant costs and benefits of the Thought Spot project.

Another limitation is the assumption that the benefits of Thought Spot as measured by the GHSQ will persist beyond the 6-month RCT period. Using this time frame assumes that 6 months is sufficient to observe expected outcomes and that any benefits observed at that time will not change (ie, increase or revert to prestudy status) after the observation and evaluation period end.

Since participants in the study are identified through self-selection, the population is limited to those who identify as having mental health concerns. The participation is voluntary and participants may withdraw at any time. Consequently, the study population may exclude segments of the population who do not identify as having mental health challenges or who may not yet recognize the need for service or support.

## Abbreviations

GHSQ: General Help-Seeking Questionnaire
ICER: incremental cost-effectiveness ratio
INB: incremental net benefit
NB: net benefit
RCT: randomized controlled trial
WTP: willingness to pay
